# The architecture of starch blocklets follows phyllotaxic rules

**DOI:** 10.1038/s41598-020-72218-w

**Published:** 2020-11-18

**Authors:** Francesco Spinozzi, Claudio Ferrero, Serge Perez

**Affiliations:** 1 Department of Life and Environmental Sciences, Polytechnic University of Marche, Ancona, Italy; 2 The European Synchrotron Radiation Facility, ESRF, Grenoble, France; 3 CNRS, CERMAV, University Grenoble Alpes, Grenoble, France

**Keywords:** Biophysics, Plant sciences, Physics

## Abstract

The starch granule is Nature’s way to store energy in green plants over long periods. Irrespective of their origins, starches display distinct structural features that are the fingerprints of levels of organization over six orders of magnitude. We hypothesized that Nature retains hierarchical material structures at all levels and that some general rules control the morphogenesis of these structures. We considered the occurrence of a «phyllotaxis» like features that would develop at scales ranging from nano to micrometres, and developed a novel geometric model capable of building complex structures from simple components. We applied it, according to the Fibonacci Golden Angle, to form several Golden Spirals, and derived theoretical models to simulate scattering patterns. A GSE, constructed with elements made up of parallel stranded double-helices, displayed shapes, sizes and high compactness reminiscent of the most intriguing structural element: the ‘blocklet’. From the convergence between the experimental findings and the theoretical construction, we suggest that the «phyllotactic» model represents an amylopectin macromolecule, with a high molecular weight. Our results offer a new vision to some previous models of starch. They complete a consistent description of the levels of organization over four orders of magnitude of the starch granule.

## Introduction

Green plants and algae produce starch for energy storage over long periods. In photosynthetic tissues, starch is synthesized in a temporary storage form during the day, since its degradation takes place at night to sustain metabolic events and energy production. For long time storage, in non-photosynthetic tissues found in seeds tubers, roots etc, the synthesis occurs in amyloplasts. The granule is well suited to this role, being insoluble in water and densely packed, but still accessible to the plant’s metabolic enzymes during germination, sprouting or regrowth.

Because it was nutritious, easy to store and carry, adapted to diverse growing conditions, and provided food, starch became a staple ingredient of the human diet for a long time and then a commodity thanks to the diversity of its functional properties. Starch is a biopolymer and consists of two major components: amylose and amylopectin. Amylose that builds up to 15–35% of the granules in most plants, is a primarily linear polysaccharide with α-(1–4)-linked d-glucose units. Amylopectin, for which a broad molecular weight in the order of 10^7^ to 10^9^ Da^1^ has been reported, is a generic term to define highly branched macromolecules in which co-exist α-(1–4)-linked d-glucose backbones and about 5% of α-(1–6)-linked branches. Such features have a profound effect on physical and biological properties, and starches obtained from different botanical origin vary in their morphology^2^ and functional properties^3^.

Starch granules occur in all shapes and sizes (spheres, ellipsoids, polygon, platelets and irregular tubules). They have diameters ranging from around 0.1 to 200 µm depending on their botanical origin. Starch granules are densely packed with semi-crystalline structures, and densities of about 1.5 g/cm^3^ have been reported^4,5^. Cryo X-ray ptychographic tomography of fully hydrated amylose-free B-type Arabidopsis leaf starch measured a density of 1.36 g/cm^3^^6^. Most of the native starch granules exhibit a Maltese cross pattern when observed under polarised light, which indicates the occurrence of crystallites having a radial orientation of their principle axis^7^. Whereas the results of several decades of intense investigations have uncovered the highly complex, hierarchical structure of the starch granule over six-orders of magnitudes, much is still unknown about its structure. At the nanoscopic level, single left-handed strands of α-(1–4)-linked glucose residues intertwine to form a parallel stranded double-helical structure having an elongation of about 5–6 nm^8,9^. Extension of these double-helices, in the range from 2.5 to 3 nm, can account for both the primary crystal lattice as well as the branching in non-random three-dimensional structures^10^.

The side-by-side association of several hundred such building elements results in the formation of densely packed crystalline platelets (or lamellae). This level of organization is well documented, both experimentally and theoretically by mathematical modelling^10^. Small-angle X-ray scattering shows a 9–10 nm periodicity which appears to be consistent across species. This feature occurs from stacks of crystalline and amorphous platelets, of which the double helix represents the former. The crystalline platelets are 4–6 nm thick. The amorphous platelets are 3–6 nm thick consisting of longer, internal segments of amylopectin chains. Transmission electron microscopy of granule fragments has led to the idea of the existence of super-helical arrangement, which would arise from an overall helical pitch to an entire amylopectin molecule. The next observable level of structural organization at the level of 100–500 nm thickness, appears in the form of rings. It is also a universal feature of starch granules, independent of their botanical origin. The rings embed both the amorphous and crystalline platelets. Alternation of two types of physical composition, densely compact shells and less dense shells, forms the growth rings. The terms “crystalline” and “amorphous/semi-crystalline” regions describe them. Any spectroscopic evidence does not firmly substantiate this terminology.

**Figure 1:**
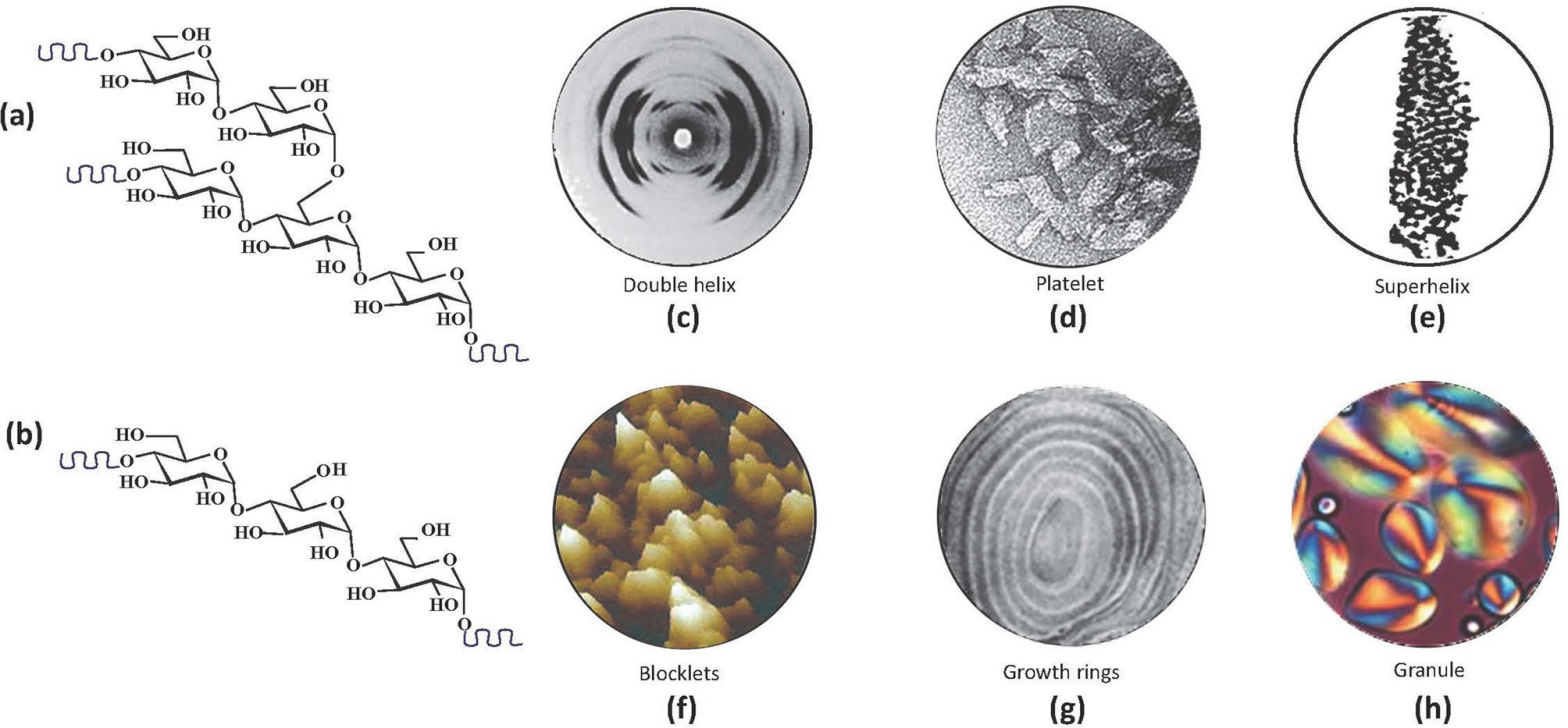
Level of the hierarchical structure of the starch granule through experimental data. Starch constituents (**a**) amylopectin and (**b**) amylose (drawn with ChemDraw).(**c**) X-ray fiber diffraction pattern indicative of a double-helical structure^53^ (**d**); Transmission Electron micrograph of crystalline platelets obtained from acid hydrolysis of starch^22^ (**e**); Transmission Electron microscopy of granule fragments (**f**); in situ AFM image technique of granular starches when exposed to iodine vapour under humid environments showing «blocklets» with a size of 10–500 nm in diameter^42^ (**g**); alternation of amorphous and semi-crystalline «growth rings» with a thickness of 100–400 nm)^54^ (**h**); extending from the hilum, Maltese cross observed under polarised light for starch granule having sizes ranging from 1 to 100 µm in diameter^55^.

Intermediary to the 9 nm repeat and the “growth ring”, a putative level of organization, referred to as “blocklets”, has been suggested by a series of direct or indirect observations. Scanning Electron Microscopy and Atomic Force Microscopy on partially digested starch granules have allowed partial visualization of the “blocklets” which has been substantiated by Small-Angle Neutron and X-ray scattering techniques^11^. They have a roughly ellipsoidal shape of 50 to 500 nm in diameter. The largest “blocklets” appear to predominate near the granule surface. Several hypotheses have suggested that these “blocklets” could be arranged in a local order that would explain the relative resistance of the outer shell of the starch granule. Because an intricate matrix of a softer material embeds the “blocklets”, it has proven to be difficult to isolate them as single macromolecular entities. Only one report mentions the observation of starch nanoparticles that could provide essential experimental evidence of “blocklets” as individually isolated particles^12^. Figure 1 depicts the levels of the hierarchical structure of the starch granule through experimental data.

The existence of **platelets** of known composition and geometry that would be organized in the form of **super-helicoidal structure** to form “**blocklets**” of the roughly ellipsoidal shape of apparent 50 to 500 nm in diameter is the only set of information available. And yet, these features are landmarks of all starch granules, irrespective of their botanical origin. **Could it be that the formation of “blocklets” obeys general rules that control the morphogenesis of the hierarchical structure of biological materials?**

### Hypotheses

To answer the question in the last sentence, we turn our attention to the field of phyllotaxy. The term phyllotaxy refers to the regular arrangements of lateral organs (leaves on a stem; florets on a composite flower head; scales on a cone axis) in plants and ferns. Such patterns around the stem are highly regular, resulting in the opposite, alternate, or spiral arrangements. It is conventional to classify these phyllotactic patterns into three broad categories: whorled, spiral, and distichous. The most widespread phyllotactic structures are spiral and distichous (alternate) if one organ is formed per node, or decussate (opposite) if two organs are formed per node. These arrangements reveal lattice-like spiral or helical structures, called parastichies. The occurrence of such apparent regularity has attracted the attention of many natural scientists and philosophers. While describing the consistency of such arrangements, mathematicians have developed computer applications capable of recreating phyllotactic patterns. Among them, spiral phyllotaxis is the most common one. The mathematical analysis of spiral phyllotaxy describes how developing organs are formed and depict how one leaf is initiated per node, with the divergence angle between successive leaves approaching the Fibonacci angle (also referred to as the golden angle) of 137.5°. Such descriptions only provide a limited elucidation of phyllotaxy. Other models have been envisaged and tested, which explore biochemical, chemical, and physical principles underlying the regulation of phyllotaxy, and more generally, the morphogenesis.

Intending to elucidate the chemical basis of morphogenesis, Turing explained how local chemical reactions could result in large-scale patterns, all being driven by a reaction–diffusion equation^13^. Patterns are formed by the interplay between 2 types of “chemical” reaction, one that activates growth (*the activator*) and one that inhibits growth (*the inhibitor*). Production of the activator stimulates further production of the *activator* but also stimulates the creation of the *inhibitor.* Several researchers^14–16^ constructed and modelled how reaction–diffusion equations could drive such developments. Two evolution equations were suggested, describing the rate of change of activator and inhibitor concentrations, which depend on the space–time coordinate. The interplay between activator and inhibitor may lead to growing periodic and symmetric patterns, satisfying functional tasks. As to whether development is guided by chemical reactions or by mechanical stresses is still an open question. The validity of the predicted model lies in comparison with the botanical observations of plant morphogenesis. This is usually performed throughout optical microscopy, which also helped to visualize those phyllotactic patterns that are observed at microscopic levels.

Although phyllotactic systems are remarkably stable, their patterns can change in the course of developmental stages or by changes occurring in Nature. Each type of phyllotactic pattern results from a particular developmental sequence. It is the result of the hierarchy of macro-levels with new emerging entities of increasing complexity. The hierarchical structure of biological materials arises from the generation of systems resulting from the accretion of subsystems during self-assembly and growth. Nature retains hierarchical material structures at all levels since most of the (bio)chemistry occurs over a limited distance. It is, therefore, logical to look for the occurrence of “phyllotaxis-like” features that would take place at a scale ranging from nano- to micrometre scales, at levels that would preface the formation of plant patterns. The determination of structures (or structure–property relationships) of biological materials, requires techniques capable of covering the range of dimensions, from nanometric to millimetric. At the nanoscale level, X-ray and neutron scatterings are the most suitable methods^17^. More specifically, these techniques are distinguished as ultra-small, small and wide-angle X-ray or neutron scattering (USAXS and USANS, SAXS and SANS, WAXS and WANS, respectively, SAS and WAS in general) as well as X-ray or neutron diffraction. Scanning microfocus techniques complement bulk experiments. Since the wavelengths of X-rays and neutrons are in the order of a few Angstroms, the atomic and molecular structure of biological materials can be detected. According to the Bragg law, *λ* = 2*d*sinθ, the smaller is the scattering angle (2θ), the larger is the correlation distance *d* (*d*-spacing) that can be determined by radiation with wavelength λ. Specifically, SAS, WAS and diffraction techniques allow access to different ranges of the scattering angles, in the order of 0.02°–5°, 3°–20° and 10°–150°, respectively, corresponding to typical *d*-spacings in the ranges 1–300 nm, 0.3–2 nm and 0.1–0.6 nm^18^.

## Results

Following our working hypothesis, we investigated how the Golden Spiral Ellipsoid could model a starch blocklet of nanometric dimensions. Throughout this article, we will refer, under the name of Golden Spiral Ellipsoid (abbreviated to as GSE), to an arrangement which is built from an assembly of ‘golden spirals’, which are made of parallelepipeds, arranged in layers of ${N}_{\mathrm{P}}$ parallelepipeds, stacked and rotated, with respect to each other, according to Fibonacci's “golden angle”. Based on the procedure described in the Method section, the size of the GSE is defined, first, by the geometry of the parallelepiped and, secondly, by the operations required to generate the GSE. In doing so, the dimensions of the parallelepipeds needed for the constructions are those of a starch crystalline component of the platelet. Those dimensions have been measured experimentally on platelets extracted from crystals resulting from the mild acid hydrolysis of starch followed by enzymic hydrolysis^14,19–22^. The platelets have shapes homothetic to the dimensions of the base plane of the unit-cell of A-type starch (*a* = 2.12 nm; *b* = 1.17 nm; *γ* = 123°). They have sizes ranging from 15 × 30 nm to 20 × 40 nm, with a width of about 6.5 nm. The 6.5 nm width can be understood based on the length of the double-helical structure perpendicular to the platelet plane. To these, a layer of 2.5 nm was added, corresponding to the amorphous component in the platelet. It was placed on top of the crystalline platelet. Such a construction would generate a molecular object having a thickness of 9.0 nm.

As detailed in the Method section, two different GSEs have been constructed, referred to as case #1 and case #2. For both cases, we have fixed the *a* to *b* ratio to *r* = 0.55 and the thickness *t* of the platelets to 9.0 nm . Prisms angles are $\gamma_{\text{P}} = 57^\circ$, $\beta_{\text{P}}  = \alpha_{\text{P}} = 52.5^\circ$. The volume fraction of the amorphous component in the platelets has been fixed to $\phi = 2.5/9.0 = 0.28$. Bottom and top heights of the blocklet are 200 nm, whereas the bottom, medium and top lengths of the basis parallelogram diagonals are 12.4, 74.5 and 12.4 nm, respectively. The only difference between the two cases is the unique tilt angle formed by the platelets with respect to the $z$ axis, which is $90^\circ$ for case #1 and $52.5^\circ$ for case #2. For both cases, the same assembling operations have been applied to both direct and specular platelets (defined in the caption of Fig. 6). Calculated molecular weight are 2.5·10^9^ (case #1) and 2.0·10^9^ Da (case #2).

**Figure 2:**
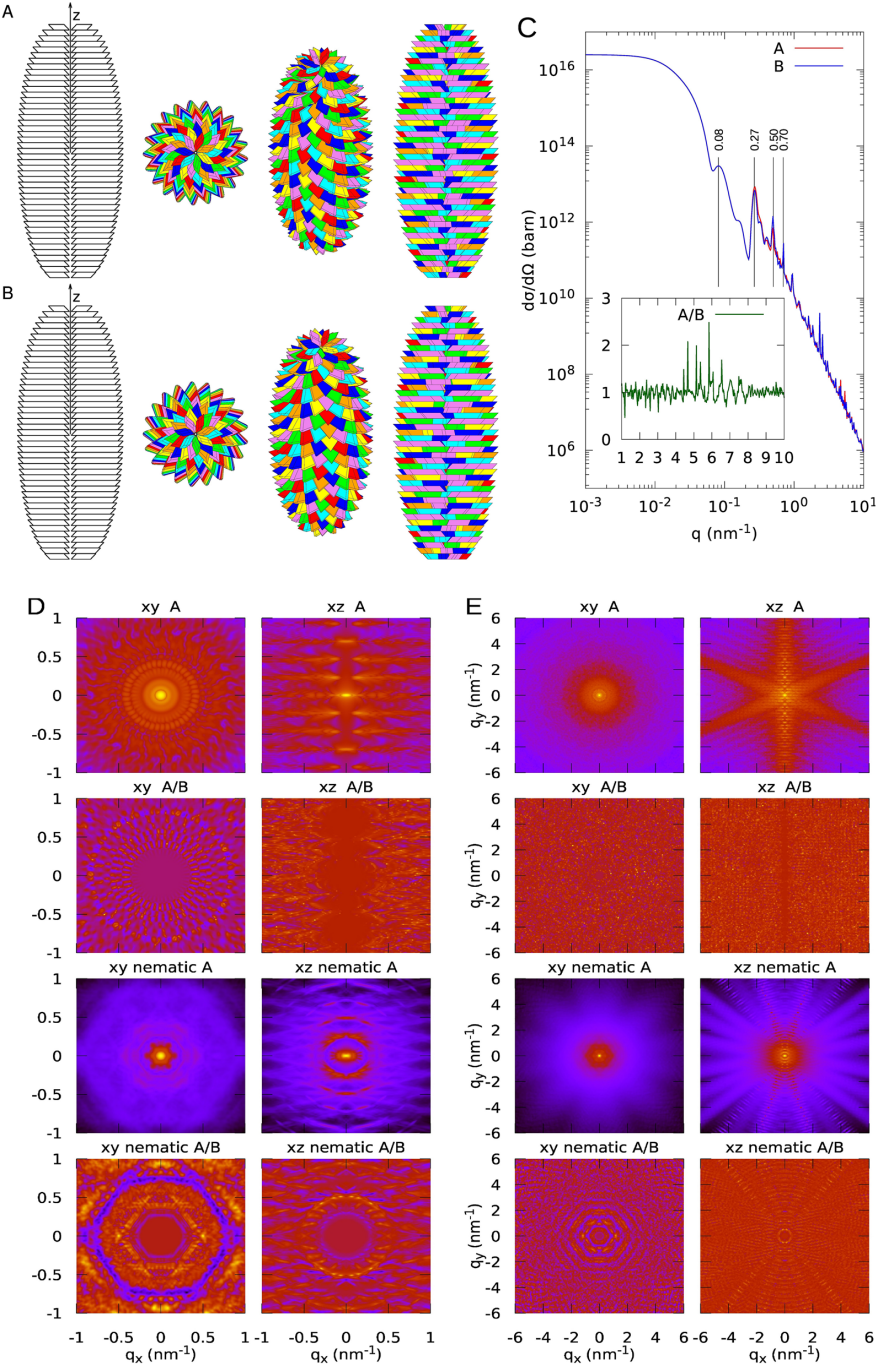
Different views of a GSE according to the present model. Case #1. Geometrical parameters are: $N_{P} = 7$, $t = 9.0$ nm, $r = 0.55$, $\gamma_{P} = 57^\circ$, $\beta_{P} = \alpha_{P} = 52.5^\circ$, $\nu = 0$, $\phi = 0.28$, $R_{b} = R_{m} = R_{t} = 1.1$ nm, $L_{b} = L_{t} = 200$ nm, $d_{b} = d_{t} = 12.4$ nm, $d_{m} = 74.5$ nm, $\beta_{b} = \beta_{m} = \beta_{t} = 90^\circ$. The number of layers in both bottom and top truncated cones is 23, and the volume fraction is $c_{V} = 0.46$. The overall molecular weight of the GSE is $M_{bl} = 2.5$ GDa. A. The first view on the left shows the inner cavity: platelets are not rotated by ${\uptheta }_{F}$ but only stacked for clarity. The other three pictures from left to right have been drawn by rotating the GSE along the $x$ axis by 0°, 45° and 90°, respectively. The platelets of each of the seven spirals are represented with the same colour: red, orange, yellow, green, cyan, blue and violet. B. GSEs obtained with the same parameters of panel A but with the specular images of the parallelepiped. C. SAXS and WAXS (highlighted in the inset) isotropic differential scattering cross-section of a GSE with direct and specular platelets, corresponding to panels A and B, respectively. D. Two-dimensional SAXS maps of the GSEs represented in panel A or B. The X-ray beam is assumed to be along the $z$ axis. The $xy,$ and $xz$ views refer to the GSE with its long axis parallel or perpendicular to the X-ray or neutron beam. The first four maps refer to the GSE fixed in the space. The last four maps refer to GSEs arranged according to a nematic order, with order parameter $S = 0.8$, assuming that the director of the nematic phase is parallel or perpendicular to the X-ray or neutron beam ($xy$ nematic and $xz$ nematic, respectively). E. WAXS maps corresponding to panel D.

**Figure 3:**
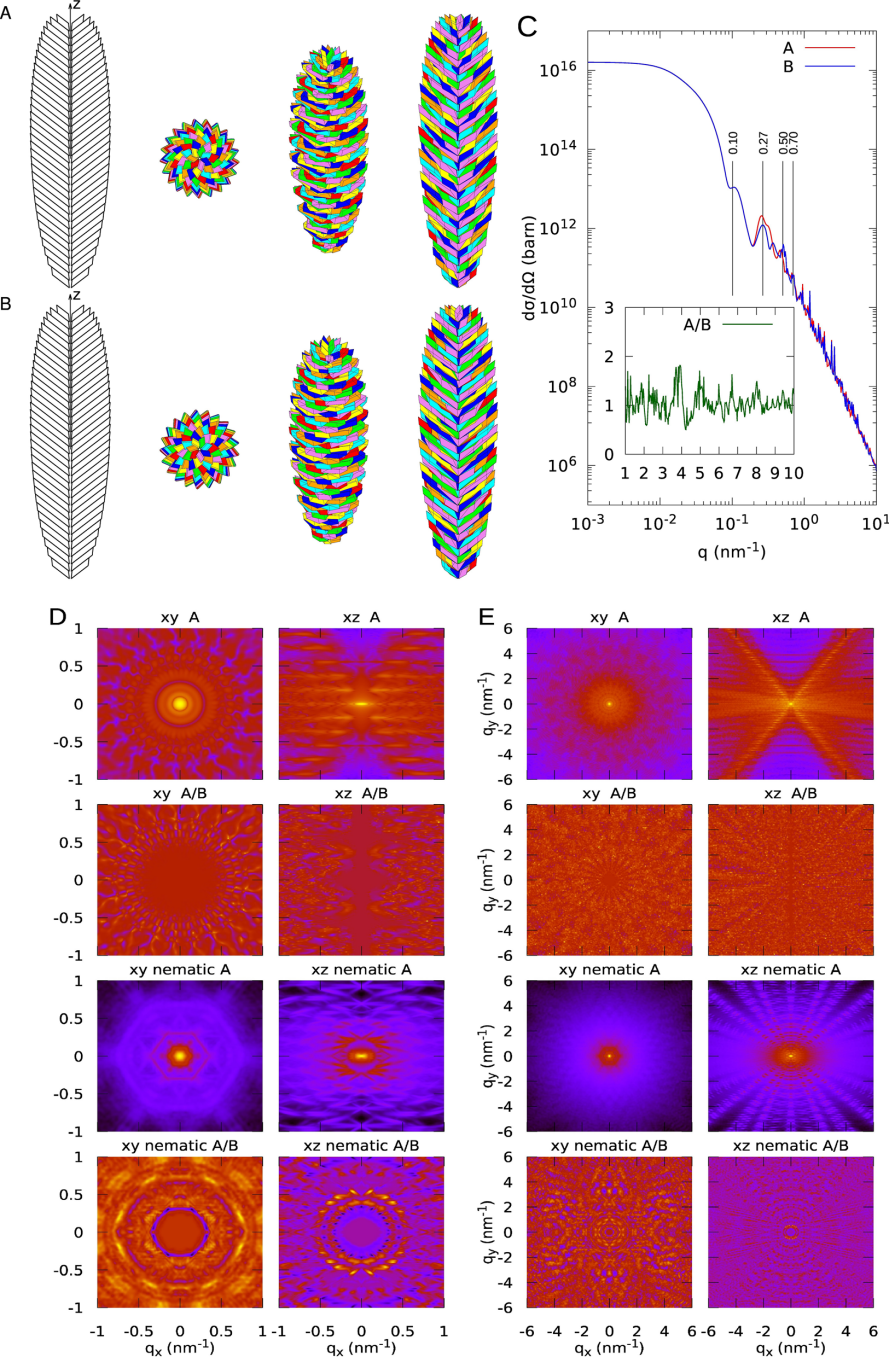
Different views of a GSE according to the present model. Case #4. (3.23)Geometrical parameters are: $N_{P} = 7$, $t = 9.0$ nm, $r = 0.55$, $\gamma_{P} = 57^\circ$, $\beta_{P} = \alpha_{P} = 52.5^\circ$, ${\upnu } = 0$, $\phi = 0.28$, $R_{b} = R_{m} = R_{t} = 1.1$ nm, $L_{b} = L_{t} = 200$ nm, $d_{b} = d_{t} = 12.4$ nm, $d_{m} = 74.5$ nm, $\beta_{b} = \beta_{m} = \beta_{t} = 52.5^\circ$. The number of layers in both bottom and top truncated cones are 18, and the volume fraction is $c_{V} = 0.63$. The overall molecular weight of the GSE is $M_{bl} = 2.0$ GDa. (**A**) The first view on the left, shows the inner cavity: platelets are not rotated by ${\uptheta }_{F}$ but only stacked for clarity. The other three pictures from left to right have been drawn by rotating the GSE along the $x$ axis by 0°, 45° and 90°, respectively. The platelets of each of the seven spirals are represented with the same colour: red, orange, yellow, green, cyan, blue and violet. (**B**) GSEs obtained with the same parameters of panel A but with the specular images of the parallelepiped. (**C**) SAXS and WAXS (highlighted in the inset) isotropic differential scattering cross-section of a GSE with direct and specular platelets, corresponding to panels A and B, respectively. (**D**) Two-dimensional SAXS maps of the GSEs represented in panel A or B. The X-ray beam is assumed to be along the $z$ axis. The $xy$. and $xz$ views refer to the GSE with its long axis parallel or perpendicular to the X-ray or neutron beam. The first four maps refer to the GSE fixed in the space. The last four maps refer to GSEs arranged according to a nematic order, with order parameter $S = 0.8$, assuming that the director of the nematic phase is parallel or perpendicular to the X-ray or neutron beam ($xy$ nematic and $xz$ nematic, respectively). (**E**) WAXS maps corresponding to panel D.

We have first calculated the X-ray isotropic differential scattering cross-section, $d\sigma /d{\Omega }\left( q \right)$ seen in Eq. (13), corresponding to randomly oriented blocklets. These two-dimensional profiles are reported in a wide range of the modulus $q$ ($q = \left( {4{\uppi }/{\uplambda }} \right)\sin {\uptheta }$), corresponding to SAXS (low $q$, up to approximatively 5 nm^−1^) and WAXS (high $q$, from approximatively 5 to 10 nm^−1^). According to the results of^17^ for waxy maize starch, in all cases, the excess scattering length densities (SLDs) of amorphous and crystalline components have been fixed to $\Delta \rho_{{{\text{am}}}} = 4.97 \cdot 10^{10}$ cm^−2^ and $\Delta \rho_{\text{cr}} = 6.07 \cdot 10^{ - 12}$ cm^−2^, corresponding to electron densities of amorphous starch, crystalline starch and water of 514, 554 and 337 e nm^−3^ respectively. Results are shown in Figs. 2 and 3, panel C. As expected, in the SAXS region, up to ~ 0.1 nm^−1^, direct and specular platelets cannot be distinguished, whereas, at high $q$, small but significative differences between the two objects arise, as highlighted in the insets of Figs. 2 and 3, panel C, where the ratios between direct and specular WAXS signals are plotted. Interestingly, only for case #2 but not for case #1, the most significant variations of $d\sigma /d{\Omega }\left( q \right)$ between direct and specular conditions fall in the broad peak at *q* = 0.27 nm^−1^ (Fig. 2, panel C). To note, the position of this peak corresponds to $\theta_{F} /t$, suggesting that it represents the helical pitch of the spiral. In both Figs. 2 and 3 we indicate the positions of other peaks. The one at 0.08–0.1 nm^−1^ is in the typical first broad peak of the form factor of an ellipsoid with semi-axes in the order of 80, 80 and 200 nm. The peaks at 0.5 nm^−1^ and at 0.7 nm^−1^ agree with $2\pi /c$ and $2\pi /t$ , respectively, indicating repeat distances along the chain direction and in the direction perpendicular to the platelets. All the other diffraction peaks, not highlighted, are due to the complex interplay of all the geometrical features of the GSE with two SLD levels platelets.

We have then calculated, based on Eq. (14), X-ray anisotropic differential scattering cross-sections (three-dimensional SAXS and WAXS maps) of blocklets fixed in the laboratory reference system. According to the two canonical configurations, we have assumed that the incoming X-ray (which is supposed to travels along the axis of the laboratory reference system, with the modulus of the wave vector $k_{0} = 2\pi /\lambda$) is parallel or perpendicular to the longest $z$ axis of the blocklet (and *xy* and *xz* views, respectively). The wavelength $\lambda$ has been fixed to 0.1 nm. For SAXS, data have been calculated in the $q_{x}$ and $q_{y}$ range from $- 1$ to $1$ nm^−1^; for WAXS, the range has been extended from $- 6$ to $6$ nm^−1^. Notice that, considering the Ewald sphere, the value of the $q_{z}$ depends on $q_{x}$ and $q_{y}$, i.e. $q_{z} = k_{0} \left( {\cos \left( {\sin^{ - 1} \left( {q_{x} /\left( {k_{0} \cos \left( {\tan^{ - 1} \left( {q_{y} /q_{x} } \right)} \right)} \right)} \right)} \right) - 1} \right)$. SAXS and WAXS maps, for the cases #1 and #2, are reported in Figs. 2 and 3, panels D, top four images (SAXS), and panels E, top four images, (WAXS).

Finally, we have calculated three-dimensional SAXS and WAXS maps of GSEs forming a nematic phase (Eq. 14), assuming that the director of the phase is oriented in the direction parallel or perpendicular to the X-ray or neutron beam ($xy$ and $xz$ nematic views, respectively). In all calculations, the order parameter $S$ has been fixed to 0.8, indicating a relatively high degree of nematic order. Interestingly, despite the randomization along the longest axis of the blocklet, the nematic order allows distinguishing between the direct and the specular order, as we can appreciate by a comparison of the corresponding $xz$ nematic view in the WAXS maps in Figs. 2 and 3, panels D, bottom four images (SAXS), and panels E, bottom four images, (WAXS).

## Discussion

The results of the constructions of “theoretical” blocklets provide a guide-line to decipher the several partial descriptions of blocklets reported in the literature. The concept of crystalline units in starch is not new and can be traced initially back to the prescience of Nägeli^24^. It was Badenhuizen^25^ who first demonstrated the presence of natural resistant units of material in chemically degraded starch. He consequently described these resistant blocks as "blökchen Strüktur", from which derives the term "blocklet concept". This concept was re-introduced by Gallant et al.^26^, with this type of organization originally being hypothesized based on microscopic observations made by Badenhuizen in 1937. Transmission electron microscopy^27^, light microscopy^28^, the use of atomic force microscopy (AFM)^29–36^ and X ray and Neutron Small Angle Scattering^11^ have allowed for some partial visualisation of blocklets. Further evidence of the blocklets, as an intermediate level of structure, was also confirmed from several enzymatic degradation studies of starch. The structures were termed nodules or protrusions that ranged from 150 to 300 nm^29^ in the early documentation; these protrusions have been reported to consist of smaller particles measuring 20–50 nm^11,30,32,33^**.** An asymmetric structure having an axial ratio of 2:1 or 3:1 seems to characterize the overall shape of the blocklets. But there exist variations in their maximum lengths: These lengths range from 130–250 nm for pea starch^26^, to 20–50 nm for potato starch granules^35^ as opposed to the 400–500 nm reported by^26^ and 10–30 nm for corn starch granules^29^. The following summarises the consistent features that emerge despite such observed differences^37^. (1) The blocklet structure displays shape similarity but exhibits different sizes in different plants. (2) Within the same plant, a range of dimensions may occur. (3) Throughout the granule, there is a continuous occurrence of blocklets. (4) There may not be any relation between the size of the blocklet and the thickness of the growth and amorphous rings**.** (5) It is likely that within the blocklet assembly, there may be defects in the amorphous rings and assembled loosely. (6) An interconnecting matrix may surround blocklets or group of blocklets. (7) There are no continuous structures between the growth-rings and the amorphous rings.

**Figure 4:**
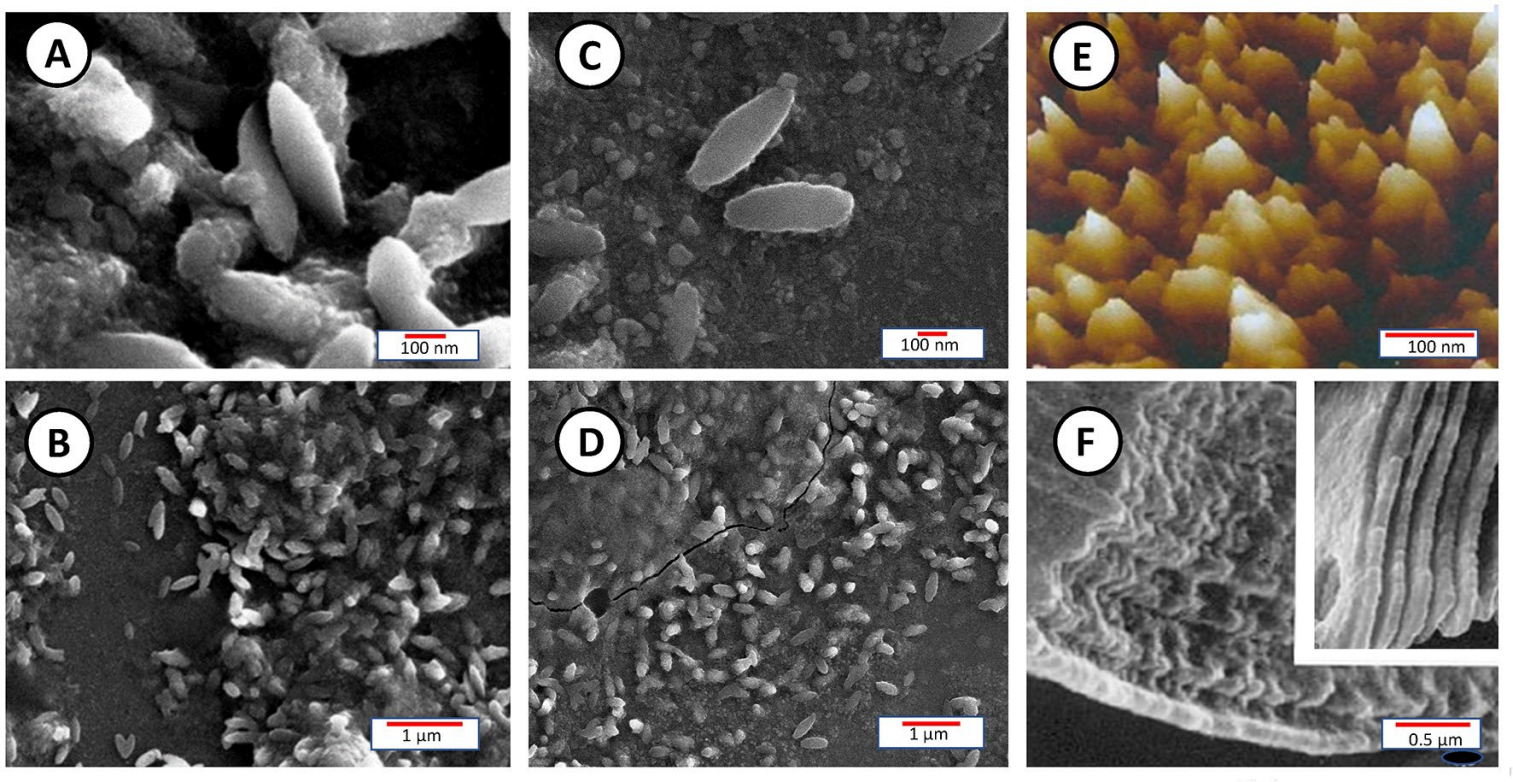
A Scanning electron micrograph of one fraction suspension of way maize collected after six days of acid hydrolysis scale bar = 100 nm; (**B**) same as in **A**; scale bar = 1 µm^41^. (**C**) Scanning electron micrograph of one fraction suspension of way maize collected after six days of acid hydrolysis scale bar = 100 nm; (**D**) same as in **C**; scale bar = 1 µm^56^; (**E**) the effect of iodine-absorption on the crystallinity of developing wheat starch granule^28^; (**F**) Scanning electron micrograph of cereal starch granule after α-amylolysis showing resistant shells composed of blocklet-like structures. Potato starch, Maize starch^57^.

There has been a long quest for extracting and isolating individual blocklets. Before that, the presence of blocklets was revealed somehow indirectly by scanning electron microscopy of cereal starch granules after α-amylolysis showing resistant shells composed of blocklet-like structures^38^ (Fig. 4A). Another observation was made while studying the effect of iodine-absorption on the crystallinity of developing wheat starch granule^36^ (Fig. 4D). It is only recently that was reported the first isolation of individual starch nanoparticles from maize obtained by mild acid hydrolysis followed by repeated water washings^12^. The Scanning Electron Microscopy image of isolated starch nanoparticles show objects in the form of flat ellipsoids, of 150 nm width and 500 nm length, with an approximated thickness of 20–30 nm. The fact that these “blocklets” were isolated as separate units would suggest that the authors had separated amylopectin molecules (Fig. 4B–F). Unfortunately, the authors did not attempt any further analytical or crystallographic characterizations to elucidate if that were true or not.

These findings are nevertheless providing converging pieces of evidence to suggest that the ellipsoid or “cigars” shape appearance of these starch nanoparticles, along with their dimensions, are reminiscent of the overall shape and dimensions of the “theoretical” blocklets. In such a model, the amylopectin macromolecule would be composed of a series of crystalline platelets + amorphous layers having a width of 9 nm. Less difference should be emphasized between the crystalline platelets and their amorphous layers. A significant proportion of the granule is in the form of helical order; ^13^C CP/MAS NMR analysis of starch granules indicates that much of the amylopectin in the crystalline-amorphous layer is in a double-helical arrangement^39^. Their intricate association of double helices and their connecting segments would represent the fundamental structural elements, having a 9 nm width periodicity.

Such fundamental elements would be stacked and rotated one with respect to each other according to the Fibonacci’s “golden angle”. The final dimensions and shape would result from the size of the initial platelets, their chirality and the number of spirals. For a blocklet, modelled by a golden ellipsoid having shapes and dimensions, displayed in Figs. 2 and 3, the molecular weight would be in the range of 10^9^ Daltons. The compactness of the packing elements constituting the blocklet is high. By analogy to the images displayed in Figs. 7 and 8**,** it may exist blocklets with smaller dimensions.

The GSE model of amylopectin may provide a detailed explanation of the work of Oostergetel and van Bruggen^40,41^ who proposed the occurrence of a super-helical structure in starch from their combined electron tomography and cryo-electron diffraction of frozen-hydrated granule fragments. They observed a helical organization which revealed an arrangement of the crystalline platelets far more complicated than was previously thought to be the case. The proposed model describes the occurrence of a continuous regular crystalline network that would form a skeleton around which the double-helical segments organize to build a continuous network of left-handed helices. The interpenetration of neighbouring helices generates a more or less continuous super-helical structure. In the context of their experimental conditions, the authors state that such a semi-crystalline structure has a diameter of approximately 18 nm and a pitch of 10 nm. Within the super helices, there would exist a central cavity having a diameter of about 8 nm. The model was further analyzed in view of microfocus synchrotron SAXS data^42^. Additional detailed data from microfocus-synchrotron SAXS and WAXS data were later provided, again on potato starch granules^43^. Although supporting the helical platelet model, these authors indicate that other organizations are possible.

In the GSE model, amylopectin is initiated from one crystalline platelet with the divergence angles between successive platelet approaching the Fibonacci angle. The resulting structure forms, by essence, a super helix for which scattering patterns can be computed.

The model of amylopectin derived from the present work may be evaluated with respect to the previous models proposed to explain the amylopectin ultrastructure and the organization of clusters of chains within the crystalline and amorphous platelet^44–47^. For historical reasons, the “cluster model” has received the most attention. It describes an arrangement where all amylopectin chains run parallel to each other with alternating amorphous and crystalline regions. The model has been developed from the study of the molecular weight of the structural studies of acid-treated starch granules. Rather little is known about the structure of the “cluster”, and most discussions are based on indirect pieces of evidence. The basic definition of the “cluster” is still missing, and the model, which cannot explain the compactness of the organization of the chains in the starch granule, cannot be constructed from molecular modelling. The “backbone model” predicts an orientation of the chain in the amorphous region perpendicular to the direction of the crystalline region. In this model, shorter chains constitute the crystalline regions, whereas the longer interconnecting chains occur in the amorphous region. This platelet organization is possible because in acid-treated starches the long chains are removed by acid entirely due to their amorphous nature. At the same time, the more crystalline sections remain intact^48^. This model, albeit being insufficient to predict the complete structure of amylopectin would be compatible with the “GSE” construction described in the present work.

With the observation that Nature retains hierarchical material structures at all levels, we have considered the occurrence of «phyllotaxis» like features that would take place at a scale ranging from nano to micrometres. To this end, we have developed a novel geometric model capable of building complex structures from simple parallelepipedic repeat units. These repeat units are translated and rotated, according to the Fibonacci Golden Angle, to form a Golden Spiral. Several Golden Spirals, typically seven, are intertwined and the size of the parallelepipeds, are modified along the axis of the spiral to form a particle with an overall ellipsoidal shape. We have then derived the theoretical model to simulate the X-ray and neutron scattering patterns. Both small-angle and wide-angle scattering processes have been considered. As a result, we have obtained the X-ray fingerprints of these new GSEs, and we have shown how they can reveal an internal arrangement of their chiral repeat units. The scale-up procedure we have exploited, from a repeat unit with relatively simple geometry to an assembly that follows a precise repetition scheme is quite general. It can be applied to investigate other macromolecular architecture in the nano to micrometric fields.

**Figure 5:**
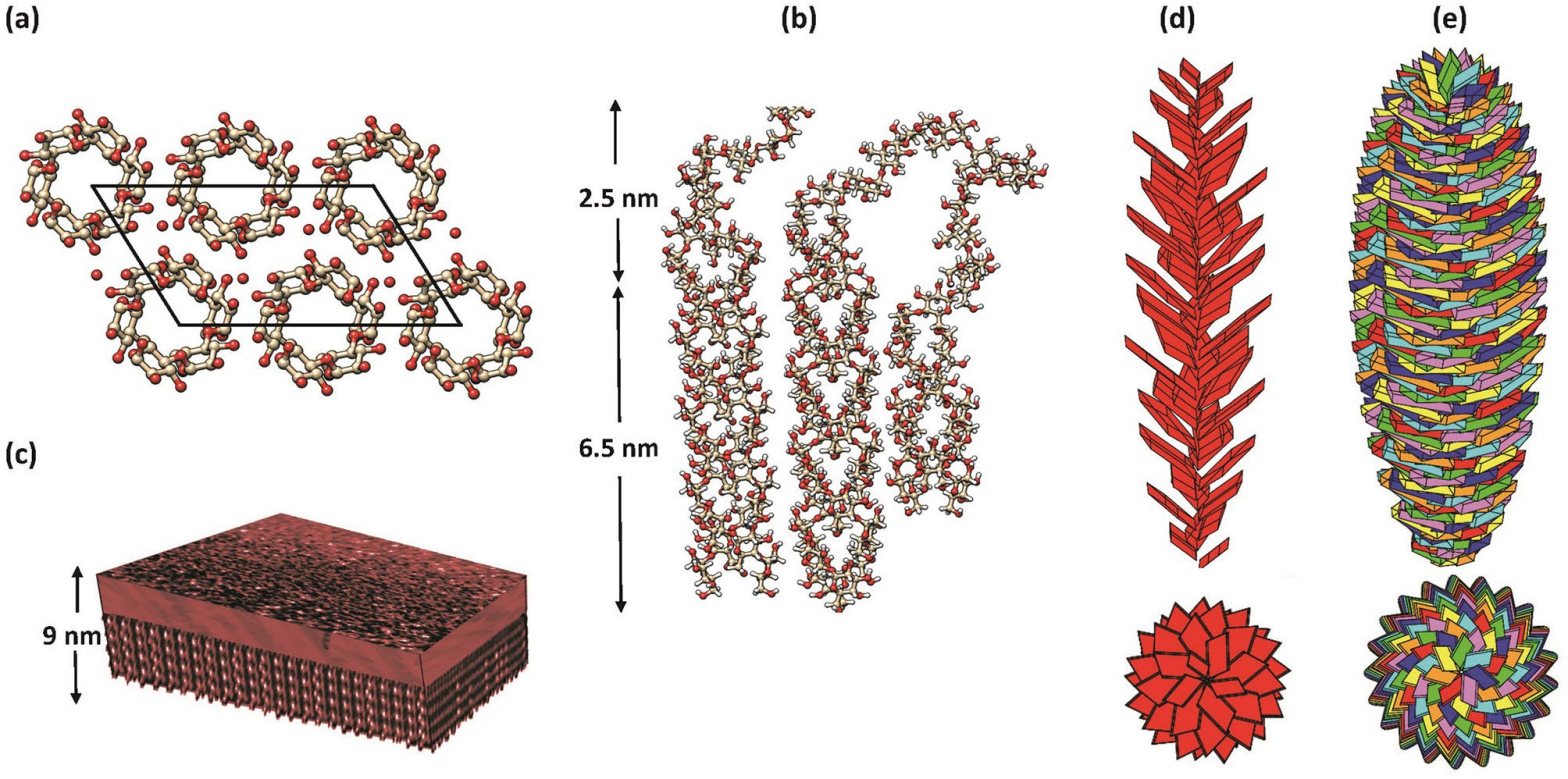
Starch: from the double helix to blocklet. (**a**) Projection of the crystalline structure of A-type starch composed of the left-handed, parallel stranded double helices, packed in a parallel fashion in a unit cell of dimensions *a* = 2.12 nm; *b* = 1.17 nm; *c* = 1.07 nm; *γ*  = 123.5°). (**b**) The first level of branching between two strands of double helices occurs without any distortion. Some spatial configuration of single amylose chains leads to bridging neighbouring double helices in packing orientations corresponding to those found in the crystal structure. The level of helical order is greater than the extent of crystalline order. The cumulative distance of these structural arrangements is about 6.5 nm of crystalline order and 2.5 nm of the less ordered constituents. This accounts for the 9 nm repeat recurrently observed in all starches. (**c**) Schematic representation of starch platelet, in agreement with the experimentally observed dimensions of nanocrystals that have 5 to 7 nm thickness, characteristic geometrical features such as 60–65° acute angles; form parallelepipedal blocks that contain several hundred of double helices. To these, a layer of 2 to 2.5 nm is added to model the embedded less ordered constituents. (**d**) Representation of one Golden Spiral built from platelets described in (**c**) stacked and rotated with respect to each other, according to Fibonacci's golden angle. The number of layers is 36. The height of the spiral is 200 nm. (**e**) Representation of one GSE made up of seven helices. The platelets of each of the seven spirals are represented with the same colour (red, orange, yellow, green, cyan, blue and violet). The height of the GSE is 200 nm, with a length of the platelet diagonal ranging from 12 nm and 74.5 nm.

We have applied the principle of our construction to the elucidation of an enigmatic level of organization of the starch granule, the so-called “blocklets”. They have estimated dimensions ranging from 20 to 500 nm and are composed by a 9 nm arrangements of amorphous and crystalline platelets made of parallel stranded double helices. The results of the constructions of “theoretical” blocklets indicate that a variety of possible structures can be formed. Among those with the highest density, appear ellipsoids forms. Their «cigars» like appearance, along with their dimensions are reminiscent of the individual blocklets which has been isolated from starch nanoparticles from maize obtained by mild hydrolysis. From the convergence between the experimental findings and the theoretical construction, we suggest that the “phyllotactic” model represents an amylopectin macromolecule with a molecular weight in the range of 10^9^ Da as a result of the compactness of the constituting elements. The present results explain the occurrence of a super-helical structure from electron tomography and cryo-electron diffraction. While establishing a viable model of a consistent hierarchical organization over four orders of magnitude (Fig. 5), the present results offer a new 3-dimensional vision to reconsider previously experimentally reported data and extend our understanding of the complexity of the structures and the underlying biosynthetic events^49,50^.

## Methods

### Method: golden spiral ellipsoid: construction and form factor

#### Principles of construction of a GSE

The method we have developed to provide a geometric construction of a GSE based on parallelepipedic prisms (hereafter depicted by the symbol P) is here described in full detail.

**Figure 6:**
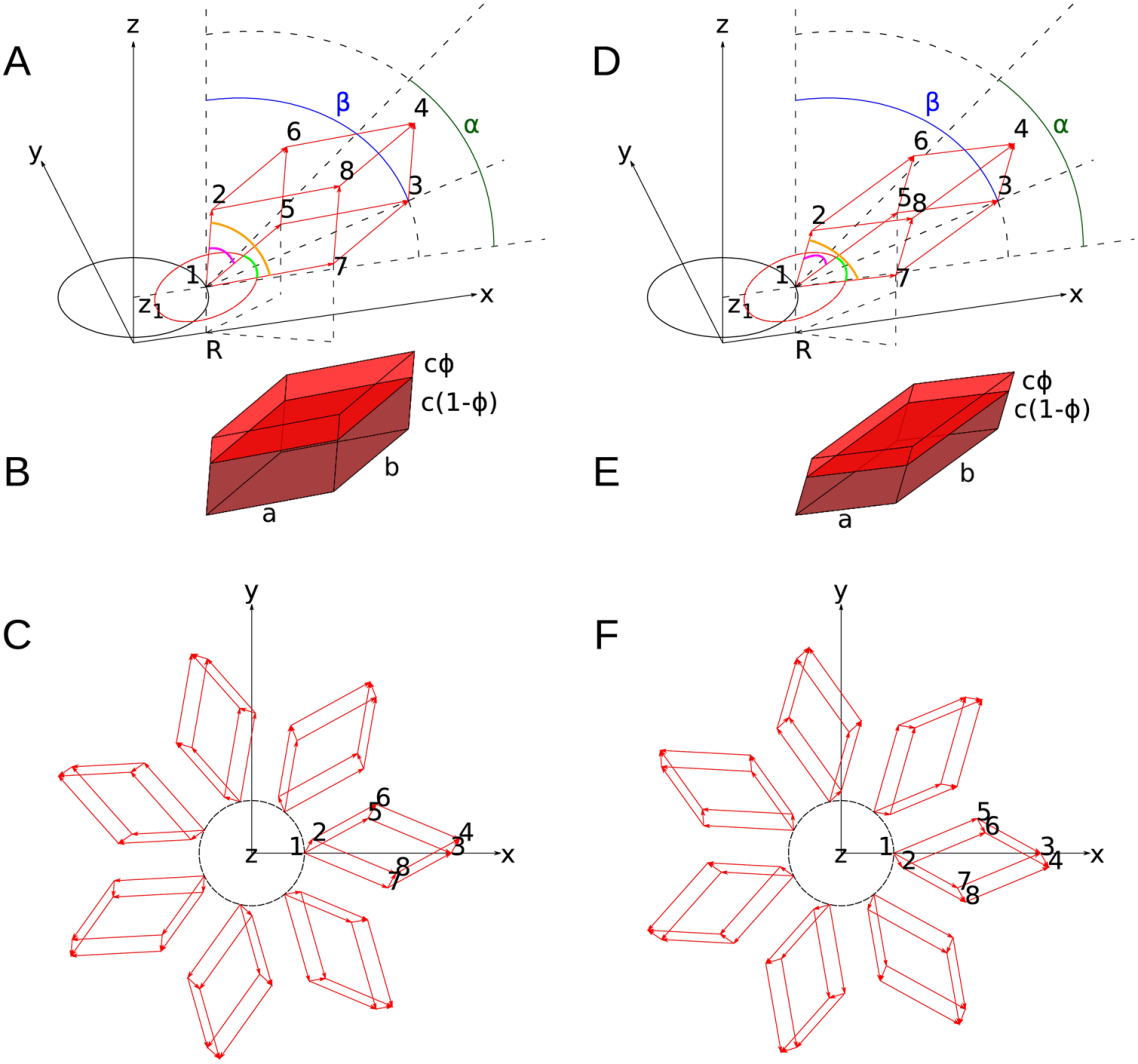
Dimensions and orientation of the parallelepiped representing one plate of a GSE formed by layers of seven platelets. (**A**) Positions and numbering of the eight vertices of the parallelepiped in the $xyz$ reference system, $z$ being the axis of the GSE. The $x,y,z$ coordinates of the vertex 1 are $R$,0,$z_{1}$. The parallelepiped angles $\alpha_{P}$, $\beta_{P}$ and $\gamma_{P}$ are represented by the magenta, the orange and the green arcs, respectively. The basis of the parallelepiped, defined by the vertices 1, 5, 3 and 7, is placed on the same plane of to the circle represented in red. $\beta$ is the angle between the $z$ axis and the diagonal of the basis parallelogram. $\alpha$ is the angle that defines the widening of the truncated cone representing the inner cavity of the GSE. (**B**) Representation of the parallelepiped shown in panel A according to the low-density and high-density (red and dark-red volumes, respectively) starch. $\phi$ is the low-to-high density volume fraction and corresponds to the ratio along the $c$ axis, as shown. (**C**) Top view of a layer of seven parallelepipeds that share the same distance $R$ of their vertex 1 from the z-axis. (**D**) Specular image of the parallelepiped shown in panel A across the $xz$ plane. Vertices 6, 8, 5 and 7 have been renumbered. (**E**) Specular representation across the $xz$. plane of the parallelepiped shown in panel D. (**F**) Top view of seven specular parallelepipeds displaced as described for panel D.

The prism is defined as a parallelepiped by three non-planar vectors ${\mathbf{a}}$,${\mathbf{b}}$ and ${\mathbf{c}}$, displaced in a right-handed sequence, with corresponding lengths *a*,*b* and *c* and unit vectors $\widehat\bf{\text{a}}$, $\widehat \bf{\text{b}}$ and $\widehat \bf {\text{c}}$. We define $\alpha_{\text{P}}$, $\beta_{\text{P}}$ and $\gamma_{\text{P}}$ the angles between two unit vectors, according to the three scalar products $\widehat \bf {\text{a}} \cdot \mathop {\widehat{\text{b}}}\limits = \user2{ }\cos \beta_{\text{P}}$_,_ $\widehat \bf{\text{b}} \cdot \mathop {\widehat{\text{c}}}\limits = \user2{ }\cos \beta_{\text{P}}$ and $\widehat \bf{\text{a}} \cdot \mathop {\widehat{\text{c}}}\limits = \user2{ }\cos \beta_{\text{P}}$. For an orthogonal reference system, where $\widehat  { \mathbf{a}}$ is along the $x$-axis and $\widehat \bf {\text{b}}$ is in the $xy$ plane, the polar angles $\alpha_{c }$ and $\beta_{c }$ of the unit vector $\widehat{\text{c}}$ can be expressed as a function of the three angles $\alpha_{\text{P}}$, $\beta_{\text{P}}$ and $\gamma_{\text{P}}$, according to the following expressions: $\alpha_{c } = \tan^{ - 1} \left( {\cos \alpha_{\text{P}} /\left( {\cos \beta_{\text{P}} \sin \gamma_{\text{P}} } \right) - \cot \gamma_{\text{P}}} \right)$ and $\beta_{{\text{c}}} =\sin^{ - 1} (\left( {\cos^{2} \alpha_{\text{P}} + \cos^{2} \beta_{\text{P}} - 2\cos \beta_{\text{P}} \cos \alpha_{\text{P}} \cos \gamma_{{\text{P}}} } \right)^{1/2} /\sin \gamma_{\text{P}} )$. We now consider a translation of the prism: if its centre is placed in the point **r**_0_, the eight vertices (see Fig. 6) are in the following positions:

1 $$\begin{aligned} {\mathbf{r}}_{1} = {\mathbf{r}}_{0} - \frac{1}{2}\left( {{\mathbf{a}} + {\mathbf{b}} + {\mathbf{c}}} \right) \quad {\mathbf{r}}_{2} = {\mathbf{r}}_{0} - \frac{1}{2}\left( {{\mathbf{a}} + {\mathbf{b}} - {\mathbf{c}}} \right) \hfill \\ {\mathbf{r}}_{3} = {\mathbf{r}}_{0} - \frac{1}{2}\left( { - {\mathbf{a}} - {\mathbf{b}} + {\mathbf{c}}} \right) \quad {\mathbf{r}}_{4} = {\mathbf{r}}_{0} - \frac{1}{2}\left( { - {\mathbf{a}} - {\mathbf{b}} - {\mathbf{c}}} \right) \hfill \\ {\mathbf{r}}_{5} = {\mathbf{r}}_{0} - \frac{1}{2}\left( { - {\mathbf{a}} + {\mathbf{b}} + {\mathbf{c}}} \right)\quad {\mathbf{r}} _{6} = {\mathbf{r}}_{0} - \frac{1}{2}\left( { - {\mathbf{a}} + {\mathbf{b}} - {\mathbf{c}}} \right) \hfill \\ {\mathbf{r}}_{7} = {\mathbf{r}}_{0} - \frac{1}{2}\left( {{\mathbf{a}} - {\mathbf{b}} + {\mathbf{c}}} \right)\quad {\mathbf{r}}_{8} = {\mathbf{r}}_{0} - \frac{1}{2}\left( {{\mathbf{a}} - {\mathbf{b}} - {\mathbf{c}}} \right) \hfill \\ \end{aligned}.$$

It is worth noting that, in general, a prism is a chiral object. In the bottom of Fig. 6, its specular image, obtained by reflecting in the $xz$ plane, is shown. We assume that the GSE is a hollow object, with the inner cavity defined by a combination of two truncated cones, one on the top of the other, which share a common circle placed in the $xy$ plane and with a common axis along the $z$ direction. The radii of the bottom, middle and top circles are $R_{\text{b}}$, $R_{\text{m}}$ and $R_{\text{t}}$, respectively. The heights of the bottom and the top truncated cones are $L_{\text{b}}$ ${\text{and }} L_{\text{t}}$, respectively. Accordingly, the tilt angles of the bottom and top truncated cones, with respect to the *xy* plane, are $\alpha_{\text{b}} = \tan^{ - 1} \left( {L_{\text{b}} /\left( {R_{\text{m}} - R_{\text{b}}}  \right)} \right)$ and $\alpha_{\text{t}} = \tan^{ - 1} \left( {L_{\text{t}} /\left( {R_{\text{t}} - R_{\text{m}} } \right)} \right)$, respectively. The parallelepipeds are arranged as $N_{\text{L}}$ layers of $N_{\text{P}}$ platelets, stacked and rotated, one with respect to each other, according to Fibonacci's “golden angle”: $\theta_{\text{F}} = \pi \left( {3 - \sqrt 5 } \right) \approx 137.51^\circ$. By referring to the parallelepipeds vertices’ numbering defined in Eq. (1) and represented in Fig. 6, we assume that: (1) all the $N_{\text{P}}$ parallelepipeds of the same layer have their edge ${\mathbf{r}}_{1}$ on the truncated cone surface, with the same $z_{1}$ component; (2) the lines connecting the ${\mathbf{r}}_{1}$ and ${\mathbf{r}}_{3}$ points of any parallelepipeds, corresponding to the diagonal of the parallelogram formed by the vector **a** and **b** (referred to as basis parallelogram), form the same angle $\beta$ with the $z$ axis; and (3) have the same lengths $a$, $b$ and $c$; (4) the projection of the vectors ${\mathbf{r}}_{1}$ and ${\mathbf{r}}_{3}$ of a parallelepipeds on the $xy$ plane fall on the same straight line that crosses the origin of the reference system. Moreover, we assume that $\beta$ might linearly change as a function of the common component $z_{1}$ of the layer. Accordingly, we define bottom, middle and top values of $\beta$, referred to as $\beta_{\text{b}}$, $\beta_{\text{m}}$ and $\beta_{\text{t}}$, respectively, which lead to the following linear equations for the bottom truncated cone,

2 $$\beta = \beta_{\text{b}} + \left( {\beta_{\text{m}} - \beta_{\text{b}} } \right)\frac{{ z_{1} + L_{\text{b}} }}{{L_{\text{b}} }}.$$

and for the top truncated cone,

3 $$\beta = \beta_{\text{m}} + \left( {\beta_{\text{t}} - \beta_{\text{m}} } \right)\frac{{ z_{1} }}{{L_{\text{t}} }}.$$

By fixing, for *all* the parallelepipeds of the GSE: (1) the ratio between the sides of the basis, $r = b/a$; (2) the thickness $t$; (3) the two angles $\gamma_{\text{P}}$ and $\beta_{c}$, we can calculate the three sides of any parallelepipeds as a function of the length of the diagonal of its basis parallelogram, $d = |{\mathbf{r}}_{3}$-${\mathbf{r}}_{1} |$, as follows: $a = d/\left( {1 + ~r^{2} + 2r\cos \gamma _{\text{P}} } \right)~^{{1/2}}$, $b = ra$, $c = t/\cos \beta_{c}$ , respectively. Accordingly, the volume of the parallelepipeds is $V = abt\sin \gamma_{\text{P}}$. Moreover, by assuming an overall ellipsoidal shape of the GSE, the diagonal of the basis of the parallelepipeds can be considered to vary with $z_{1}$ according to the following relation for the bottom truncated cone,

4 $$d = d_{\text{b}} + \left( {d_{\text{m}} - d_{\text{b}} } \right) \left[ {1 - \left( {\frac{{ z_{1} }}{{L_{\text{b}}} }} \right)^{2} } \right]^{1/2}.$$

and to the next equation for the top truncated cone,

5 $$d = d_{\text{t}} + \left( {d_{\text{m}} - d_{\text{t}} } \right) \left[ {1 - \left( {\frac{{ z_{1} }}{{L_{\text{t}} }}} \right)^{2} } \right]^{1/2}.$$

where and $d_{\text{b}}$, $d_{\text{m}}$. and $d_{\text{t}}$ represent the bottom, middle and top diagonal lengths, respectively. We also define a void layer between two subsequent layers of parallelepipeds with fixed thickness $v$, measured in the direction perpendicular to the rhomboidal bases of the lower layer.

The envelope volume, representing the overall volume displaced by the GSE, can be considered the volume occupied by the solid obtained by rotating $2\pi$ around the $z$. axis the polygon defined by the coordinates $x_{j,n}$ $z_{j,n}$ with $n = 1,2,3,4$ (see the edge numbering reported in Fig. 6) of the first plate of each $j$-layer. This volume is here calculated by the so-called shell method of the integral calculus,

6 $$\begin{aligned} V_{{{\text{env}}}} & = \nu _{{1,3;1,1}} + ~\nu_ {{N_{{L}} ,2;N_{{L,4}}}} \\ \quad & + \sum\nolimits_{{j = 1}}^{{N_{L} }} {\left( {\nu _{{j,1;j,2}} + \nu _{{j,4;j,3}} } \right)} + \sum\nolimits_{{j = 1}}^{{N_{{L - 1}} }} {\left( {\nu _{{j,2;j + 1,1}} + \nu _{{j + 1,3;j,4}} } \right)} \\ \nu _{{j,n;k,m}} & = \frac{\pi }{3}\left[ {z_{{k,m}} \left( {2x_{{k,m}}^{2} - x_{{j,n}} x_{{k,m}} - ~x_{{j,n}}^{2} } \right) - z_{{j,n}} \left( {2x_{{j,n}}^{2} - x_{{k,m}} x_{{j,n}} - x_{{k,m}}^{2} } \right)} \right]. \\ \end{aligned}$$

On the other hand, the volume of the inner cavity is simply calculated by the formula of the truncated cone,

7 $$V_{\text{cav}} = \frac{{\uppi }}{3}\left[ {L_{\text{b}} \left( {R_{\text{b}}^{2} + R_{{\text{m}}}^{2} + R_{\text{b}} R_{\text{m}} } \right) + L_{t} \left( {R_{\text{m}}^{2} + R_{\text{t}}^{2} + R_{\text{m}} R_{\text{t}} } \right)} \right].$$

We can, therefore, calculate the volume fraction of all the $N = N_{\text{P}} N_{\text{L}}$ parallelepipeds with respect to $V_{\text{env}}$ as $c_{V} = \left( {N_{\text{P}} / V_{\text{env}} } \right)$ $\mathop \sum \limits_{j = 1}^{N_{\text{L}} } V_{j}$. Moreover, the overall molecular weight of the GSE is expressed as a function of the known density $\varrho_{{\text{m}}}$, according to $M_{\text{bl}} = \varrho_{\text{m}} N_{A} N_{\text{P}}\mathop \sum \limits_{j = 1}^{{N_{\text{L}} }} V_{j}$, $N_{A}$ being Avogadro’s number.

Following the principles above mentioned, we developed computer software which allows the construction of 3-dimensional structures of any GSE resulting from the following values to be input are : ${N}_{\mathrm{P}}$, $r$, ${\gamma }_{\mathrm{P}}$, ${\beta }_{\mathrm{P}}$, ${\alpha }_{\mathrm{P}}$, ${t}_{\mathrm{b}}$, ${v}$, ${R}_{\mathrm{b}}$, ${R}_{\mathrm{m}}$, ${R}_{\mathrm{t}}$, ${L}_{\mathrm{b}}$, ${L}_{\mathrm{t}}$, ${d}_{\mathrm{b}}$, ${d}_{\mathrm{m}}$, ${d}_{\mathrm{t}}$, ${\beta }_{\mathrm{b}}$, ${\beta }_{\mathrm{m}}$, ${\beta }_{\mathrm{t} }$ , which follow the above mentioned definitions.

**Table:** 

		Unit	Case #1	Case #2	Case #3	Case #4
**Geometrical parameters**						
$N_{\text{P}}$	Number of parallelepipeds *per* layer		7	7	7	7
$r$	Gatio *b*/*a*		0.55	0.55	0.5	0.5
$\gamma_{\text{P}}$	Angle between **a** and **b**	°	57	57	40	40
$\beta_{{\text{P}} }$	angle between **a** and **c**	°	52.5	52.5	60	60
$\alpha_{\text{P}}$	Angle between **b** and **c**	°	52.5	52.5	72	72
$t$	Thickness of the parallelepipeds in the direction perpendicular to the plane formed by **a** and **b**	nm	9	9	9	9
$v$	Thickness of the empty space between two parallelepipeds in the direction perpendicular to the plane formed by **a** and **b**	nm	0	0	2.6	3.9
$R_{\text{b}}$	Radius of the bottom circle	nm	1.1	1.1	4.3	3.3
$R_{\text{m}}$	Radius of the middle circle	nm	1.1	1.1	8.7	13
$R_{\text{t}}$	Radius of the top circle	nm	1.1	1.1	4.3	3.3
$L_{\text{b}}$	Height of bottom truncated cone	nm	200	200	43	130
$L_{\text{t}}$	Height of top truncated cone	nm	200	200	69	390
$d_{\text{b}}$	Diagonal of the basis bottom parallelogram	nm	12.4	12.4	35	52
$d_{\text{m}}$	Diagonal of the basis middle parallelogram	nm	74.5	74.5	43	65
$d_{\text{t}}$	Diagonal of the basis top parallelogram	nm	12.4	12.4	26	39
$\beta_{{\text{b}} }$	Angle between the z-axis and the direction of the basis bottom parallelogram	°	90	52.5	120	60
$\beta_{{{\text{m}} }}$	Angle between the z-axis and the direction of the basis middle parallelogram	°	90	52.5	90	45
$\beta_{\text{t}}$	Angle between the z-axis and the direction of the basis top parallelogram	°	90	52.5	9	36
**Scattering parameters**						
$\phi$	Amorphous to total volume fraction		0.28	0.28	0.28	0.28
$\Delta \rho_{{{\text{am}}}}$	Difference between the SLD of amorphous starch region and water	$10^{10}$ cm^−2^	4.97	4.97	4.97	4.97
$\Delta \rho_{\text{cr}}$	Difference between the SLD of semi-crystalline starch region and water	$10^{10}$ cm^−2^	6.07	6.07	6.07	6.07

**Figure 7:**
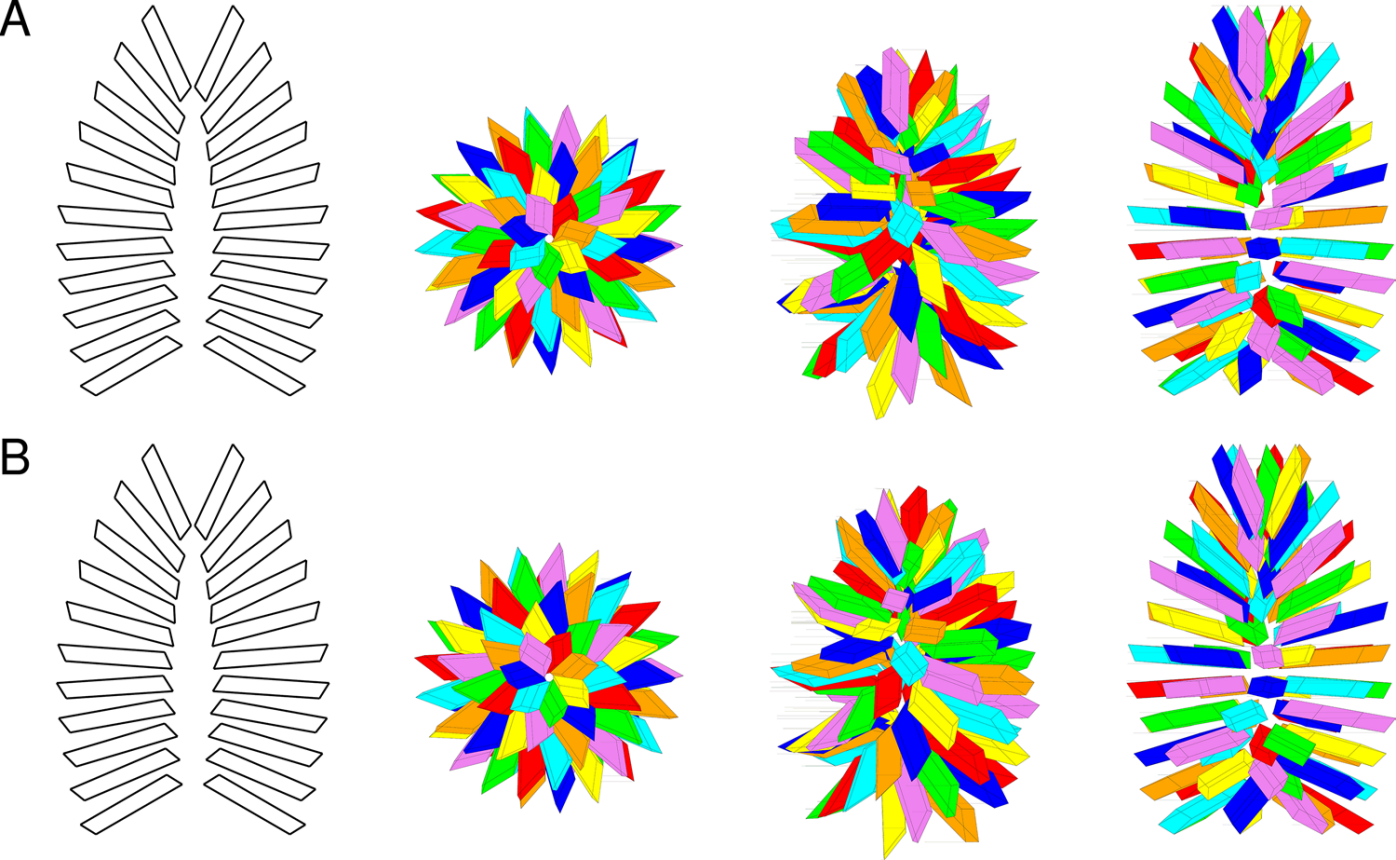
Different views of a GSE according to the present model. Case #3. Geometrical parameters are: ${N}_{P}=7$, $r=1/2$, ${\gamma }_{P}=2\pi /9$, ${\beta }_{P}=\pi /3$, ${\alpha }_{P}=2\pi /5$, $t=9.0$ nm, $\upnu =2.6$ nm, ${R}_{b}={R}_{t}=4.3$ nm, ${R}_{m}=8.7$ nm, ${L}_{b}=43$ nm, ${L}_{t}=69$ nm, ${d}_{b}=35$ nm, ${d}_{m}=43$ nm, ${d}_{t}=26$ nm, ${\beta }_{b}=2\pi /3$, ${\beta }_{m}=\pi /2$, ${\beta }_{t}=\pi /20$. The numbers of layers in the bottom and top truncated cones are 5 and 6, respectively, and the volume fraction is ${c}_{V}$ = 0.14. The overall molecular weight of the GSE is ${M}_{bl}=0.3326$  GDa. (**A**) The first view, on the left, shows the inner cavity: platelets are not rotated by ${\uptheta }_{F}$ but only stacked for clarity. The other three views from left to right have been drawn by rotating the GSE along the $x$ axis by 0°, 45° and 90°, respectively. The platelets of each of the seven spirals are represented with the same colour: red, orange, yellow, green, cyan, blue and violet. (**B**) GSEs obtained with the same parameters of panel A but with the specular images of the parallelepiped.

**Figure 8:**
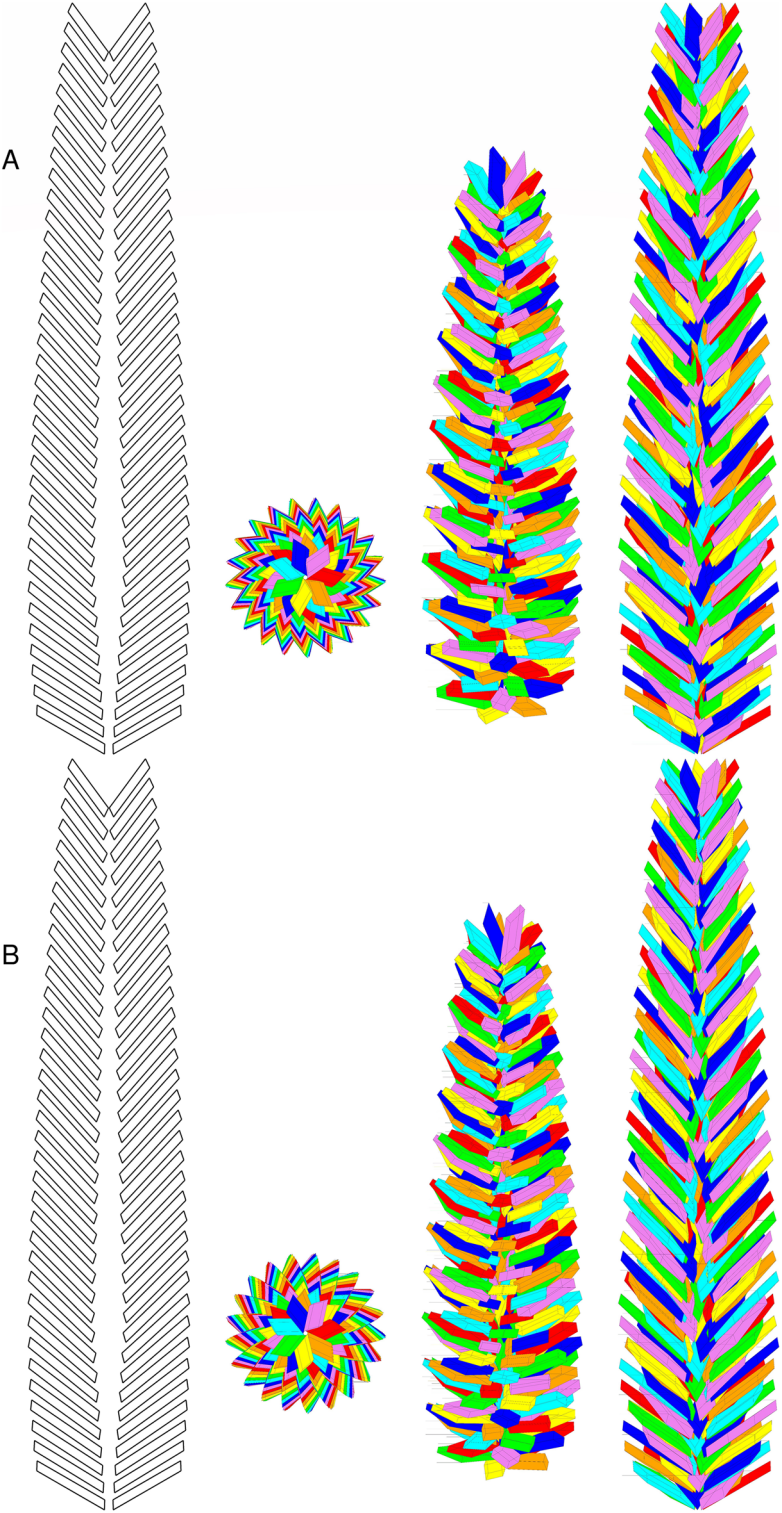
Different views of a GSE according to the present model. Case #4. Geometrical parameters are: ${N}_{P}=7$, $r=1/2$, ${\gamma }_{P}=2\pi /9$, ${\beta }_{P}=\pi /3$, ${\alpha }_{P}=2\pi /5$, $t=9.0$ nm, $\nu =3.9$ nm, ${R}_{b}={R}_{t}=3.3$ nm, ${R}_{m}=13$ nm, ${L}_{b}=130$ nm, ${L}_{t}=390$ nm, ${d}_{b}=52$ nm, ${d}_{m}=65$ nm, ${d}_{t}=39$ nm, ${\beta }_{b}=\pi /3$, ${\beta }_{m}=\pi /4$, $\beta_{t} = \pi /5$. The numbers of layers in the bottom and top truncated cones result 10 and 24, respectively, and the volume fraction is $c_{V}$ = 0.22. The overall molecular weight of the GSE is $M_{bl} = 2.2$ GDa. (**A**) The first view, on the left, shows the inner cavity: platelets are not rotated by $\theta_{F}$ but only stacked for clarity. The other four views from left to right have been drawn by rotating the GSE along the $x$ axis by 0°, 45° and 90°, respectively. The platelets of each of the seven spirals are represented with the same colour: red, orange, yellow, green, cyan, blue and violet. (**B**) GSEs obtained with the same parameters of panel A but with the specular images of the parallelepiped.

Figures 7 and 8 displays two GSEs which have been generated with the set of parameters reported in the corresponding captions.

According to the model previously introduced, the size of the GSE results, firstly, from the geometry of the parallelepiped and, secondly, from the symmetry operations that define the GSE. If the operations are applied to the specular image of the parallelepiped, a different GSE is obtained. But it is not the specular image of the object obtained by the assembly of the direct parallelepiped. The dimensions of the constructed GSEs, only result from the size and the chirality of the initial dimensions of the parallelepipeds and the number of spirals.

#### Form factor of the GSE

We have calculated the electron density and, generally by also including neutron scattering, the scattering length density (SLD), within the parallelepiped^51,52^. Since the starch is constituted by amorphous (am) and semi-crystalline (cr) regions, two excess scattering length density contrasts with respect to the bulk are defined, $\Delta \rho_{\text{am}}$ and $\Delta \rho_{\text{cr}}$, respectively. We assume that the amorphous region is at the top part along the ${\mathbf{c}}$ direction and that its volume fraction, with respect to the parallelepiped volume, is defined as $\phi$. Accordingly, the lengths of the amorphous and the semi-crystalline regions along ${\mathbf{c}}$ will be $c\phi$ and $c\left( {1 - \phi } \right)$, respectively (Fig. 6B,E). Thus, the whole parallelepiped is split into two stacked platelets, the amorphous and the semi-crystalline ones, with their centres in the positions ${\mathbf{r}}_{0{\text{am}}} = {\mathbf{r}}_{0} + {\mathbf{c}}\frac{1 - \phi }{2}$ and ${\mathbf{r}}_{0{\text{cr}}} = {\mathbf{r}}_{0} - {\mathbf{c}}\frac{\phi }{2}$, respectively. On this basis, the X-ray or neutron scattering amplitude of the whole parallelepipeds is the following Fourier transform,

8 $$A\left( {\mathbf{q}} \right) = \Delta \rho _{\text{am}} \int\limits_{{\text{V}}_{\text{am}} } e ^{{i{\mathbf{q}} \cdot {\mathbf{r}}}} d{\mathbf{r}} + \Delta \rho _{\text{cr}} \int\limits_{{V_{\text{cr}} }} e ^{{i{\mathbf{q}} \cdot {\mathbf{r}}}} d{\mathbf{r}},$$

where ${V}_{\mathrm{am}}$=$\phi V$ is the volume of the top parallelepipeds, ${V}_{\mathrm{cr}}$=$(1-\phi )V$ is the volume of the bottom parallelepipeds, $V=\mathbf{a}\cdot \mathbf{b}\times \mathbf{c}$ being the whole platelet volume, and $\mathbf{q}$ is the scattering vector, i.e. the difference between the wave vector scattered at the angle $2\theta$ and the wave vector of the incident beam. Considering the geometry figof the parallelepipeds, Eq. (2) becomes

9 $$\begin{aligned} A\left( {\mathbf{q}} \right) &= \left( {{\widehat{\mathbf{a}}} \cdot {\widehat{\mathbf{b}}} \times {\widehat{\mathbf{c}}}} \right)\left[ {\Delta \rho _{{{\text{am}}}} e^{{i{\mathbf{q}} \cdot {\mathbf{r}}_{{0{\text{am}}}} }} ~\mathop \smallint \limits_{{ - \frac{a}{2}}}^{{\frac{a}{2}}} dx_{a} ~\mathop \smallint \limits_{{ - \frac{b}{2}}}^{{\frac{b}{2}}} dx_{b} ~\mathop \smallint \limits_{{ - \frac{{c\phi }}{2}}}^{{\frac{{c\phi }}{2}}} dx_{c} ~e^{{i{\mathbf{q}} \cdot \left( {x_{a} {\widehat{\mathbf{a}}}~ + x_{b} {\widehat{\mathbf{b}}} + x_{c} {\widehat{\mathbf{c}}}} \right)}} } \right. \\ &\quad \left. { + \Delta \rho _{\text{cr}} e^{{i{\mathbf{q}} \cdot {\mathbf{r}}_{{0{\text{cr}}}} }} ~\mathop \smallint \limits_{{ - \frac{a}{2}}}^{{\frac{a}{2}}} dx_{a} ~\mathop \smallint \limits_{{ - \frac{b}{2}}}^{{\frac{b}{2}}} dx_{b} ~\mathop \smallint \limits_{{ - \frac{{c\left( {1 - \phi } \right)}}{2}}}^{{\frac{{c\left( {1 - \phi } \right)}}{2}}} dx_{c} ~e^{{i{\mathbf{q}} \cdot \left( {x_{a} {\widehat{\mathbf{a}}}~ + x_{b} {\widehat{\mathbf{b}}} + x_{c} {\widehat{\mathbf{c}}}} \right)}} } \right] \\ \end{aligned}.$$

The analytical solution is

10 $$\begin{aligned} A\left( {\mathbf{q}} \right) & = V j_{0} \left( {{\mathbf{q}} \cdot {\mathbf{a}}/2} \right) j_{0} \left( {{\mathbf{q}} \cdot {\mathbf{b}}/2} \right)[\Delta \rho_{{{\text{am}}}} \phi e^{{i{\mathbf{q}} \cdot {\mathbf{r}}_{{0{\text{am}}}} }} j_{0} \left( {\phi {\mathbf{q}} \cdot {\mathbf{c}}/2} \right) \\ &\quad + \Delta \rho_{{{\text{cr}}}} \left( {1 - \phi } \right) e^{{i{\mathbf{q}} \cdot {\mathbf{r}}_{{0{\text{cr}}}} }} j_{0} \left( {\left( {1 - \phi } \right) {\mathbf{q}} \cdot {\mathbf{c}}/2} \right)] \\ \end{aligned}.$$

where $j_{0} \left( x \right) = \sin x/x$ is the zeroth order Bessel function of fractional order.

According to the geometrical model of the parallelepipeds previously introduced, its scattering amplitude is the sum of the amplitudes of all its the *N* parallelepipeds,

11 $$A_{\text{pc}} \left( {\mathbf{q}} \right) = \mathop \sum \limits_{j = 1}^{N} e^{{i{\mathbf{q}} \cdot {\mathbf{r}}_{j,0} }} A_{{j}} \left( {\mathbf{q}} \right) .$$

where $A_{{j}} \left( {\mathbf{q}} \right)$ is calculated on the basis of Eq. (3) with ${\mathbf{a}}_{j} = {\mathbf{r}}_{j,5} - {\mathbf{r}}_{j,1}$, ${\mathbf{b}}_{j} = {\mathbf{r}}_{j,7} - {\mathbf{r}}_{j,1}$, ${\mathbf{c}}_{j} = {\mathbf{r}}_{j,2} - {\mathbf{r}}_{j,1}$ and, according to Eq. (1), ${\mathbf{r}}_{j,0} = {\mathbf{r}}_{j,1} + \left( {{\mathbf{a}}_{j} + {\mathbf{b}}_{j} + {\mathbf{c}}_{{\text{j}}} } \right)/2$. The corresponding microscopic differential scattering cross section is the squared modulus of the scattering amplitude,

12 $$\frac{d\sigma }{{d{\Omega }}}\left( {\mathbf{q}} \right) = |A_{\text{pc}} \left( {\mathbf{q}} \right)|^{2}.$$

If the GSEs are randomly oriented, the isotropic scattering cross-section is calculated by the orientational average of the vector ${\mathbf{q}}$, i.e. by the average over its polar angles $\alpha_{q}$ and $\beta_{q}$,

13 $$\frac{d\sigma }{{d{\Omega }}}\left( q \right) = \frac{1}{{4{\uppi }}}\mathop \smallint \limits_{0}^{{2{\uppi }}} d\alpha_{q} \mathop \smallint \limits_{0}^{{\uppi }} |A_{\text{pc}} \left( {\mathbf{q}} \right)|^{2} \sin \beta_{q} d\beta_{q}.$$

To note, the isotropic scattering cross-section only depends on the modulus $q$ of the scattering vector. We also consider the case of GSEs arranged in the space with a nematic order, with a preferential orientation of their long axis along the “director” of the phase. The director is defined, within the laboratory reference system, by two polar angles $\alpha_{\text{d}}$ and $\beta_{\text{d}}$. In these conditions, the anisotropic scattering cross-section is obtained by averaging over the Euler angles $\alpha$, $\beta$ and $\gamma$ that define the overall orientation of the GSEs,

14 $$\frac{d\sigma }{{d{\Omega }}}\left( {\mathbf{q}} \right) = \frac{1}{{8{\uppi }^{2} }}\mathop \smallint \limits_{0}^{{2{\uppi }}} d\alpha \mathop \smallint \limits_{0}^{{\uppi }} \sin \beta d\beta \mathop \smallint \limits_{0}^{{2{\uppi }}} d\gamma P\left( \beta \right)|{\mathbb{R}}\left( {\alpha_{{\text{d}}} ,\beta_{\text{d}} ,0} \right){\mathbb{R}}\left( { - \gamma , - \beta , - \alpha } \right)A_{\text{pc}} \left( {\mathbf{q}} \right)|^{2}.$$

In this equation, the symbol ${\mathbb{R}}$ represents the matrix that rotates the reference system, according to the convention of Rose^39^. Rotation matrices are applied to all the vectors ${\mathbf{r}}_{j,n}$ defining the position of any plate. $P\left( \beta \right)$ represents the probability distribution function of the angle $\beta$ formed by the long axis of the GSEs with the director of the nematic phase. A typical model for this function is based on the second-order Legendre polynomial $P_{2} \left( x \right) = \frac{3}{2}x^{2} - \frac{1}{2}$,

15 $$P\left( \beta \right) = \frac{1}{Z}{\exp}\left[ {a_{2} P_{2} \left( {\cos \beta } \right)} \right].$$

16 $$Z = \mathop \smallint \limits_{0}^{{\uppi }} \sin \beta {\exp}\left[ {a_{2} P_{2} \left( {\cos \beta } \right)} \right]d\beta.$$

where the exponent parameter $a_{2}$ is chosen in such a way to obtain a desired order parameter $S$, defined as the average value of the second-order Legendre polynomial,

17 $$S = \left\langle {P_{2} \left( {\cos \beta } \right)} \right\rangle = \frac{1}{Z}\mathop \smallint \limits_{0}^{{\uppi }} \sin \beta P_{2} \left( {\cos \beta } \right){\exp}\left[ {a_{2} P_{2} \left( {\cos \beta } \right)} \right]d\beta.$$

## Data Availability

The Fortran code developed to build the GSE and its scattering features is available upon request. The atomic coordinates used to construct the three-dimensional representations of A-type starch crystal structure, branching of double helices by α 1–6 linkage and the full atom representation of the content of the crystalline moiety of a platelet are available at polysac3db.cermav.cnrs.fr. Molecular graphics images displayed in Fig. 5a, b, were produced using the UCSF Chimera package from the Resource for Biocomputing, Visualization, and Informatics at the University of California, San Francisco (supported by NIH P41 RR-01081). Pettersen, E.F., Goddard, T.D., Huang, C.C., Couch, G.S., Greenblatt, D.M., Meng, E.C., and Ferrin, T.E. "UCSF Chimera—A Visualization System for Exploratory Research and Analysis." *J. Comput. Chem.* **25**(13):1605–1612 (2004).
